# Primary pelvic hydatid cyst: A rare case presenting with obstructive uropathy

**DOI:** 10.1016/j.ijscr.2018.09.044

**Published:** 2018-10-05

**Authors:** Neeraj Kumar, Raghav Garg, Ratnakar Namdeo

**Affiliations:** Dept. of General Surgery, VMMC & SJH, New Delhi, India

**Keywords:** Albendazole, Case report, Echinococcus, Hydatid, Hydronephrosis, Pelvic

## Abstract

**Introduction:**

Hydatid disease is caused by infection with larva of the cestode echinococcus. Humans are accidental intermediate hosts. Primary pelvic hydatid cyst is a rare entity.

**Presentation of case:**

This is a case report of a 50 year old gentleman who presented to us as a case of suprapubic swelling and difficulty in micturition. General physical examination and radiological evaluation confirmed a multiloculated pelvic swelling. Patient was subjected to laparotomy which confirmed the diagnosis of a primary pelvic hydatid disease.

**Discussion:**

Surgery is the optimal treatment of the pelvic hydatid disease. Radical resectional procedure or en bloc resection that removes the entire pericyst is the surgical technique of choice.

**Conclusion:**

Familiarity with atypical manifestations of hydatid disease may be helpful in making a prompt and accurate diagnosis. Surgery is the treatment of choice.

## Highlights

• Primary pelvic hydatid cysts are uncommon, usually presenting with pressure effects on the adjacent organs such as the urinary bladder and rectum.
• Combination of preoperative albendazole therapy, surgery and postoperative albendazole therapy is a useful regime.
• Familiarity with atypical manifestations of hydatid disease may be helpful in making a prompt and accurate diagnosis.

## 1 Introduction

*Work has been reported in line with the SCARE criteria*

Hydatid disease or echinococcosis is a zoonotic parasitic disease caused by genus echinococcus and family Taeniidae. There are three known forms of hydatid disease in humans: (a) cystic echinococcosis caused by *E. granulosus* (b) alveolar echinococcosis caused by *E. multilocularis* and (c) polycystic echinococcosis caused by *E. vogeli* or *E. oligarthus* [1].

Humans are accidental, intermediate hosts and become infected after ingesting parasite’s eggs. Dogs, foxes, wolves and jackals are definitive hosts passing eggs of parasites in the faeces. The cysts are mostly found in the liver (59–75%) and lung (27%), but they can be found in any part of the body [2].

Primary pelvic hydatid cysts are uncommon, usually presenting with pressure effects on the adjacent organs such as the urinary bladder and rectum. We are presenting a case of primary pelvic hydatid cyst disease with obstructive uropathy.

## 2 Case report

A 50 year old man presented with complaints of suprapubic swelling and difficulty in micturition for the last 4 months. He had to strain to pass urine and the flow was poor. Patient also complained of constipation for the last 2 months. No history of fever, vomiting, hematuria and bleeding per rectum.

On examination he had pulse rate of 86/min and blood pressure – 110/76 mmHg. General physical examination was within normal limit. Abdominal examination revealed a smooth, firm, slightly tender, nonmobile lump in suprapubic region reaching approx. 5 cm above pubic symphysis, lower limit not palpable. On digital rectal examination, a smooth spherical mass was felt anteriorly and laterally outside the rectal wall, rectal mucosa was normal.

Investigations revealed haemoglobin of 12.4 g/dl, total leucocyte count of 8900/mm^3^, platelet count of 2.54 lakh/mm, blood urea 33 mg/dl, serum creatinine 1.12 mg/dl, and serum electrolytes were normal. Liver function test showed serum bilirubin of total – 0.75 mg/dl, S.G.O.T. – 16 U/L, S.G.P.T. – 36 U/L, and alkaline phosphatase – 88 U/L. Urine examination showed pus cells (5–6/hpf) but the urine culture was sterile. Chest X-ray and ECG were normal.

Ultrasound examination revealed a cystic mass in the pelvis suggestive of a hydatid cyst with bilateral hydroureteronephrosis more on right side as compared with left. Liver and spleen were normal.

**Fig. 1:**
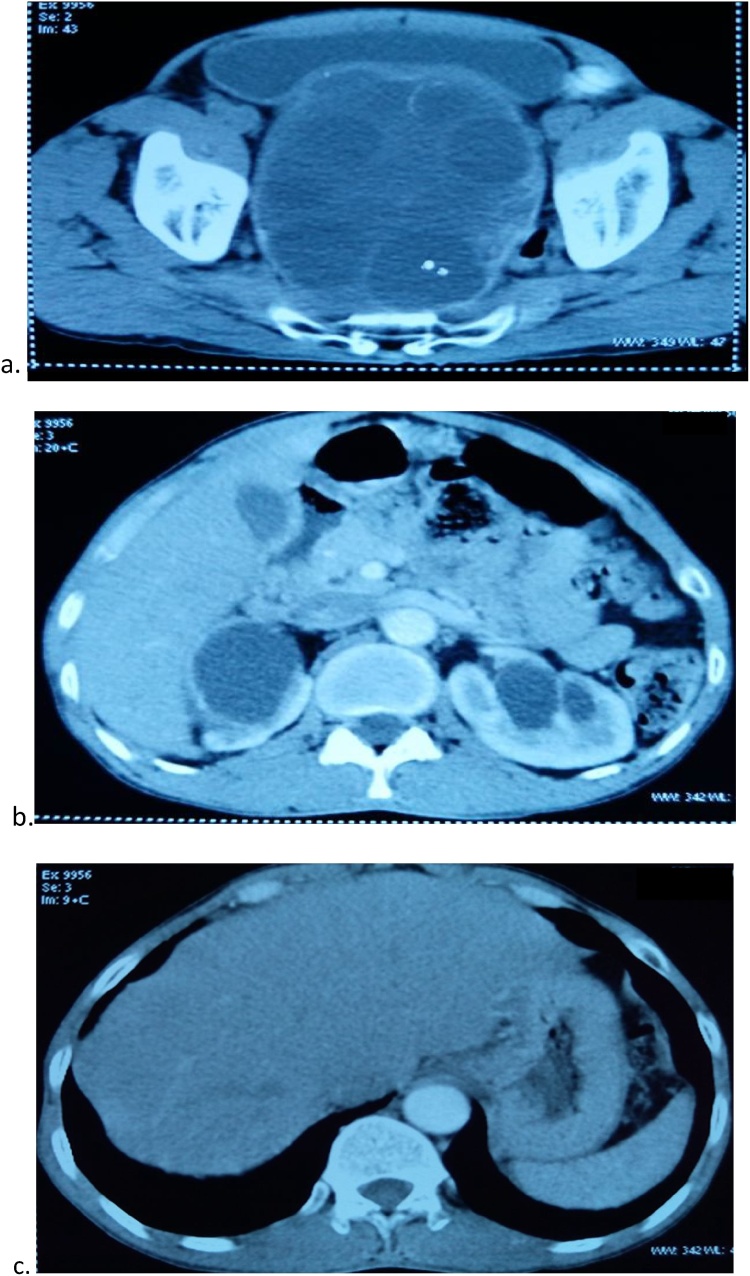
**a.** CECT abdomen and pelvis showing heterogenous hypodense lesion with multiple daughter cysts within likely hydatid cyst. **b.** showing moderate hydronephrosis on right side and mild hydronephrosis on left side. **c.** showing normal liver and spleen.

Computerized tomographic scan was suggestive of well - defined capsulated heterogeneously within, compressing the urinary bladder and rectosigmoid and reaching till the pelvic side walls – likely hydatid cyst, moderate hydronephrosis on right side and mild hydronephrosis on left side with dilated and tortuous both ureters (Fig. 1a–c).

**Fig. 2:**
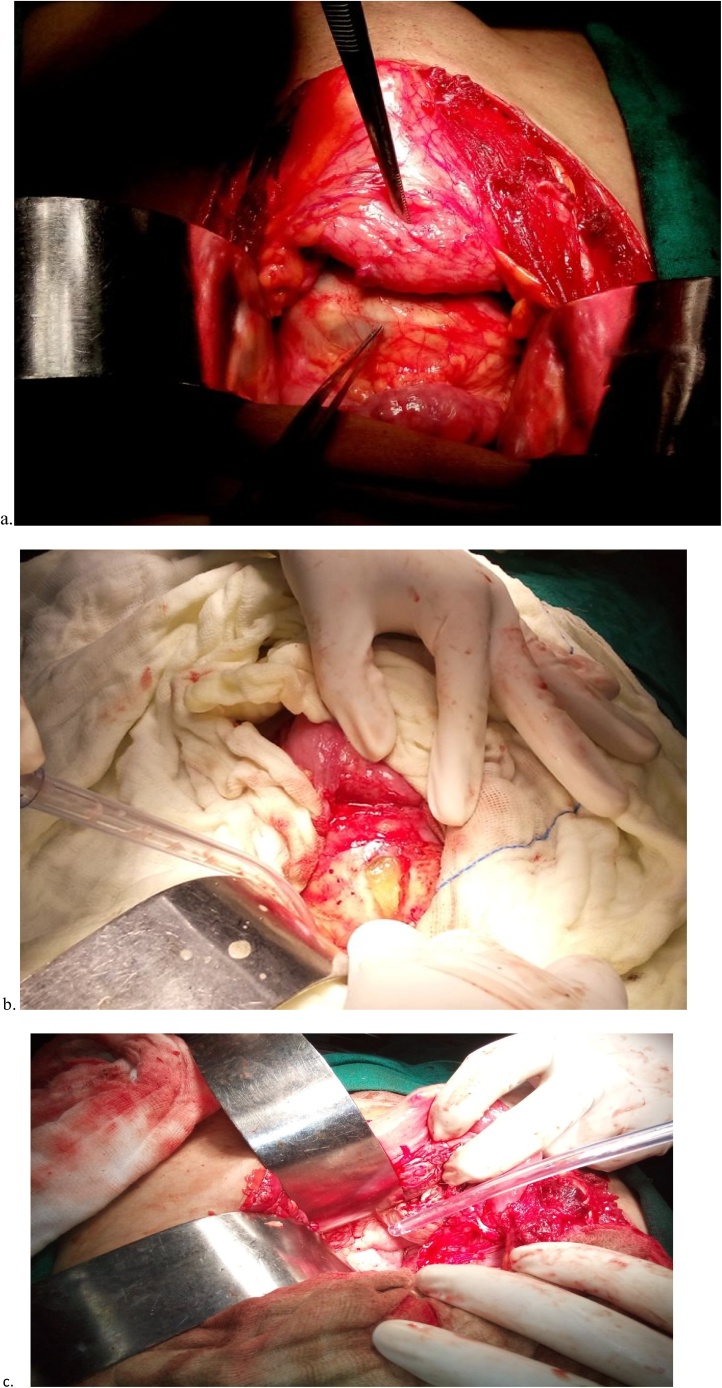
**a**. Intraoperative image showing a large, tense hydatid cyst in the pelvic cavity. **b.** shows opened hydatid cyst after its isolation by packing surrounding area with 0.5% cetrimide soaked sponges. **C.** after removing hydatid cyst.

Patient was put on one cycle of preoperative albendazole therapy (10–15 mg/kg/day) for 28 days. Exploratory laparotomy was done and liver, spleen, mesentery, omentum were found to be normal. A large tense hydatid cyst was noted in the pelvic cavity, densely adhered to urinary bladder, sigmoid mesocolon, rectum and iliac vessels laterally. Upper part of cyst was separated anteriorly from the urinary bladder and on left side from sigmoid colon and mesocolon. After mobilization hydatid cyst was isolated by packing surrounding area with 0.5% cetrimide soaked sponges and cyst opened under controlled condition. All daughter cysts and laminated membrane removed completely (Fig. 2a, b). The part of ectocyst which was densely adherent to vital neighbouring structures could not be removed. A drain was placed in pelvis and abdomen closed in layers. Final diagnosis was confirmed by histopathological examination.

Postoperative period was uneventful and patient was discharged on 5^th^ postop day. Patient was put on 3 cycles of albendazole therapy. Each cycle of albendazole therapy was of 28 days duration. After each cycle patient was advised a gap period of 2 weeks, and in that period liver function test and complete blood counts were assessed and found to be normal. Patient was symptom free after 6 months of follow up.

## 3 Discussion

Hydatid disease is caused by infection with larva of the cestode echinococcus. Humans are accidental intermediate hosts. The hydatid disease is endemic in areas such as Mediterranean countries, the Middle East, South America, Australia, New Zealand, Turkey and Central Asia particularly China, where people are in close contact with sheep and dogs [3]. No sexual predominance is recognized. Hydatid cystic disease is commonly seen in younger adults although the infestation is acquired in childhood during contact with infected animals [3].

Liver is the most common site involved, followed by lungs, kidney, bone and brain. Other sites such as heart, spleen, pancreas, omentum, ovaries, pelvis, orbit and muscles are rarely affected. Usually pelvic hydatid cysts are secondary to the rupture of liver or spleen hydatid cyst. Primary pelvic hydatid cysts are rare with an incidence between 0.2–2% [4], because in its natural life cycle the parasite must pass through filters of liver and lungs. A solitary cyst in the pelvic cavity can be considered primary only when no other cysts are present.

Primary pelvic hydatid cyst’s pathophysiology is not clearly established. There are theories that in such a case the hydatid embryo gains entry to the pelvis either by hematogenous or lymphatic route [5]. There are no specific clinical features of primary pelvic hydatid cyst, often presents as an unusual pelvic mass with pressure effects on adjacent organs such as urinary bladder, rectum, uterus, ovaries, fallopian tubes and lumbosacral nerve plexus leading to clinical features like obstructive uropathy (as in this case), renal failure, constipation, obstructed labour, menstrual irregularities, infertility [4], foot drop [6].

Routine laboratory investigations are usually normal except in cases presenting with renal failure. Various serological tests are also available but they must be used and interpreted in correlation with epidemiological data, clinical features and especially imaging investigations.

The first line imaging is the ultrasonography. Ultrasound feature of pelvic hydatid cyst are similar to hepatic cyst [7]. Classification proposed by Gharbi [7] can be accepted for other locations. CECT abdomen and pelvis gives more precise information regarding size, location, adherence to neighbouring structures, and number of the cysts as well as an excellent depiction of the visceral organs involvement. MRI has some advantages over CT scan in the evaluation of the postsurgical residual lesions and recurrences.

Surgery is the optimal treatment of the pelvic hydatid disease. Radical resectional procedure or en bloc resection that removes the entire pericyst is the surgical technique of choice. Partial cystectomy or tissue sparing procedures are alternative surgical procedures in cases where the ectocyst is densely adhered to the surrounding vital structures and its removal can be more harmful [8]. Preoperative use of albendazole therapy facilitates the surgery by reducing the intracystic pressure, whereas postoperative albendazole therapy decreases the risk of recurrences of hydatid cystic disease. Combination of preoperative albendazole therapy, surgery and postoperative albendazole therapy is a useful regime. A correct preoperative diagnosis is very important, so that all the steps are taken to prevent spillage during surgery. Moreover a preop course of albendazole therapy can also be given.

## 4 Conclusion

The primitive localization of the hydatid cyst in the pelvis is rare, its clinical symptomatology may be confusing. Familiarity with atypical manifestations of hydatid disease may be helpful in making a prompt and accurate diagnosis. Surgery is the treatment of choice. But prevention is the best way to reduce the incidence of this pathology.

## Conflicts of interest

None.

## Funding

This research did not receive any specific grant from funding agencies in the public, commercial, or not-for-profit sectors.

## Ethical approval

Not required. Exempted from our institute.

## Consent

Written informed consent was obtained from the patient for publication of this case report and accompanying images. A copy of the written consent is available for review by the

Editor-in-chief of this journal on request.

## Author contribution

Dr. Neeraj Kumar was the chief operating surgeon in the case. All the authors were involved in patient management in the post-operative period. Dr. Neeraj Kumar, Dr. Raghav Garg, Dr. Ratnakar Namdeo equally contributed in collection of data from the preoper-ative, intraoperative and postoperative periods and in drafting the case report. Dr. Neeraj Kumar and Dr. Raghav Garg further contributed by editing and proof reading the report and gave its final form for submission. All the authors had read and approved the final report.

## Registration of research studies

Not Required.

## Guarantor

Dr. Raghav Garg.
